# Diagnostic value of multimodal ultrasound imaging in differentiating benign and malignant TI-RADS category 4 nodules

**DOI:** 10.1007/s10147-019-01397-y

**Published:** 2019-03-01

**Authors:** Shufang Pei, Shuzhen Cong, Bin Zhang, Changhong Liang, Lu Zhang, Juanjuan Liu, Yuping Guo, Shuixing Zhang

**Affiliations:** 1 The Second School of Clinical Medicine, Southern Medical University, Guangzhou, Guangdong People’s Republic of China; 2 Department of Ultrasound, Guangdong Academy of Medical Sciences/Guangdong General Hospital, Guangzhou, Guangdong People’s Republic of China; 3 Department of Radiology, the First Affiliated Hospital of Jinan University, Guangzhou, Guangdong People’s Republic of China; 4 Department of Radiology, Guangdong Academy of Medical Sciences/Guangdong General Hospital, No. 106 Zhongshan Er Road, Guangzhou, 510080 Guangdong People’s Republic of China

**Keywords:** Multimodal ultrasound Imaging, Superb microvascular imaging, Real-time elastography, Thyroid nodule

## Abstract

**Background:**

Differential diagnosis of benign and malignant thyroid imaging reporting and data system category 4 (TI-RADS-4) nodules can be difficult using conventional ultrasound (US). This study aimed to evaluate whether multimodal ultrasound imaging can improve differentiation and characterization of benign and malignant TI-RADS-4 nodules.

**Methods:**

Multimodal ultrasound imaging, including US, superb microvascular imaging (SMI), and real-time elastography (RTE), were performed on 196 TI-RADS-4 nodules (78, benign; 118, malignant) in 170 consecutive patients. The sensitivity, specificity, accuracy, false negative rate (FNR), and false positive rate (FPR) of each single method and that of multimodal US imaging were determined by comparison with surgical pathology results.

**Results:**

The sensitivity, specificity, accuracy, FNR, and FPR for US were 65.25%, 69.23%, 66.84%, 34.75%, 30.77%, respectively; for SMI were 77.97%, 93.59%, 84.18%, 22.03%, 6.41%, respectively; RTE, 80.51%, 84.62%, 82.14%, 19.49%, 15.38%; and for multimodal US imaging were 94.08%, 87.18%, 91.33%, 6.93%, 12.82%, respectively. The areas under the received operating characteristic curve for US, SMI, RTE, and multimodal US imaging in evaluating benign and malignant TI-RADS-4 nodules were 67.2%, 84.40%, 86.60%, and 95.50%, respectively.

**Conclusions:**

The initial clinical results suggest that multimodal US imaging improves the diagnostic accuracy of TI-RADS-4 nodules and provides additional information for differentiating malignant and benign nodules.

## Introduction

With the increased use of ultrasound (US) in medical practice, there has been an increase in the number of detected thyroid nodules, which are detected in about 20–67% of the general population [1–3]. It is, therefore, important to establish a standard method to accurately assess thyroid nodules. In May 2017, the American Academy of Radiology launched the Thyroid Imaging Reporting and Data System (TI-RADS) classification system [4], in which all thyroid nodules are divided into five categories: 1, 2, 3, 4, and 5. Categories 1–3 nodules are considered to be likely benign, and category 5 nodules are highly suspicious of being malignant. Typically, the nodule characteristics of categories 1–3 (benign) and category 5 (malignant) are obvious; therefore, the nodules can be easily diagnosed using conventional US [5, 6].

**Fig. 1:**
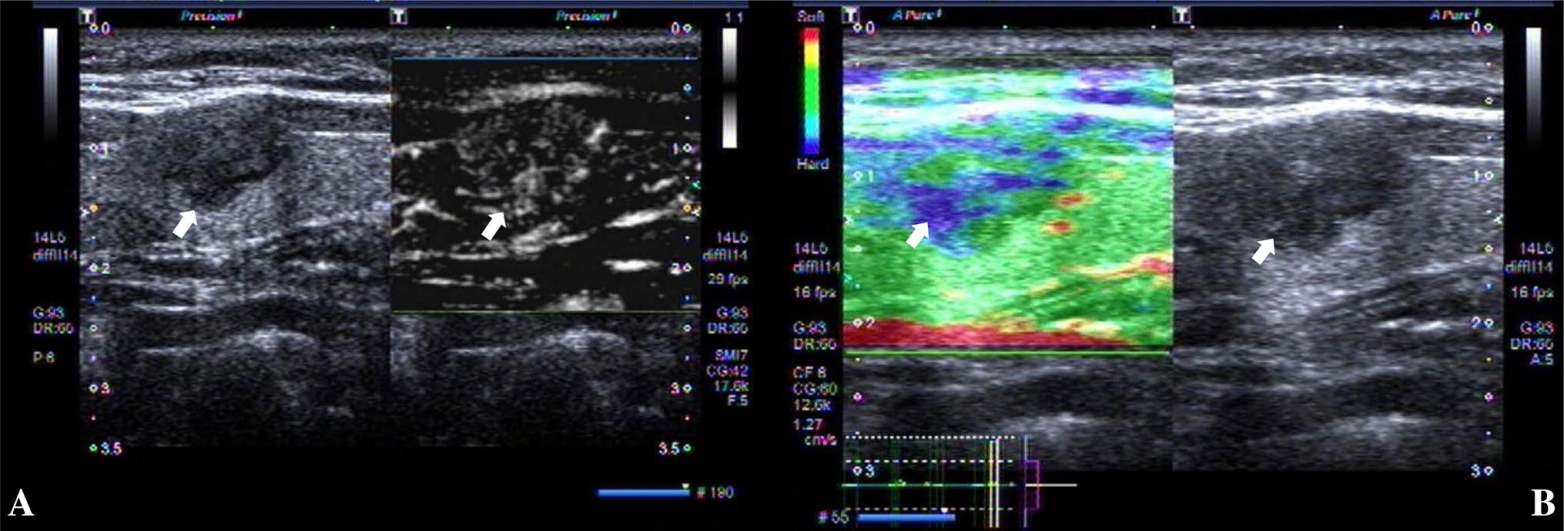
Images in a 43-year-old woman who underwent routine. A 12 mm left thyroid nodule (arrows) with solid, hypo or isoechogenicity, well defined margin, wider than tall shape, no calcification was found at conventional ultrasound and assessed as benign, ACR score of 4 and classified as TI-RADS category 4. **A** Type III was assigned at superb microvascular imaging. **B** A score of 3 was assigned at real-time elastography. This nodules was assessed as malignant at multimodal ultrasound imaging. This thyroid nodule was diagnosed as papillary thyroid carcinoma at surgery

**Fig. 2:**
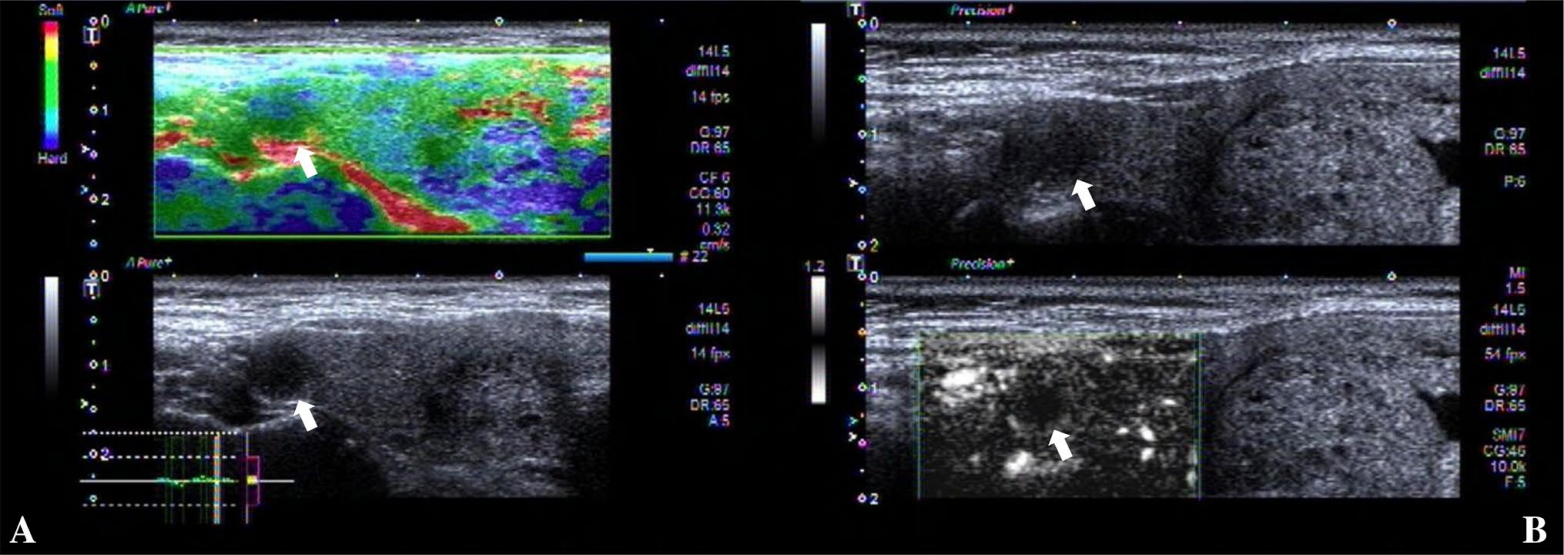
Images in a 51-year-old man who underwent routine. A 7 mm left thyroid nodule (arrows) with solid, hypoechogenicity, irregularity margin, wider than tall shape, no calcification was found at conventional ultrasound and assessed as malignant, ACR score of 6 and classified as TI-RADS category 4. **A** Type II was assigned at superb microvascular imaging. **B** A score of 2 was assigned at real-time elastography. This nodules was assessed as benign at multimodal ultrasound imaging. This thyroid nodule was diagnosed as nodular goiter at surgery

However, in contrast, differentiating benign and malignant TI-RADS category 4 nodules is difficult [7, 8]. The ultrasonographic features of these thyroid nodules are complex [9–11], and the characteristics of conventional US images often overlap. The conventional ultrasound features of some nodules were shown to be benign, and the pathology of the surgery was confirmed to be malignant (Fig. 1); while the conventional ultrasound features of some nodules showed malignancy, and the pathology confirmed by surgery was benign (Fig. 2). Frequently, there are similarities and differences in the characteristics of these diseases, which can easily lead to misdiagnosis of patients and challenges for clinicians and patients. Therefore, it is particularly important to accurately diagnosis benign and malignant nodules. However, there is no relevant research concerning these issues.

Therefore, the purpose of this study was to evaluate the diagnostic performances of multimodal US imaging techniques in differentiating benign and malignant TI-RADS category 4 nodules. In an effort to improve the diagnostic accuracy for such nodules and facilitate clinical management, we investigated the following imaging techniques: conventional US, superb microvascular imaging (SMI), and real-time elastography (RTE).

## Materials and methods

The institutional review board approved this retrospective study and waived the requirement for patient approval or informed consent for the review of patient images and records.

**Fig. 3:**
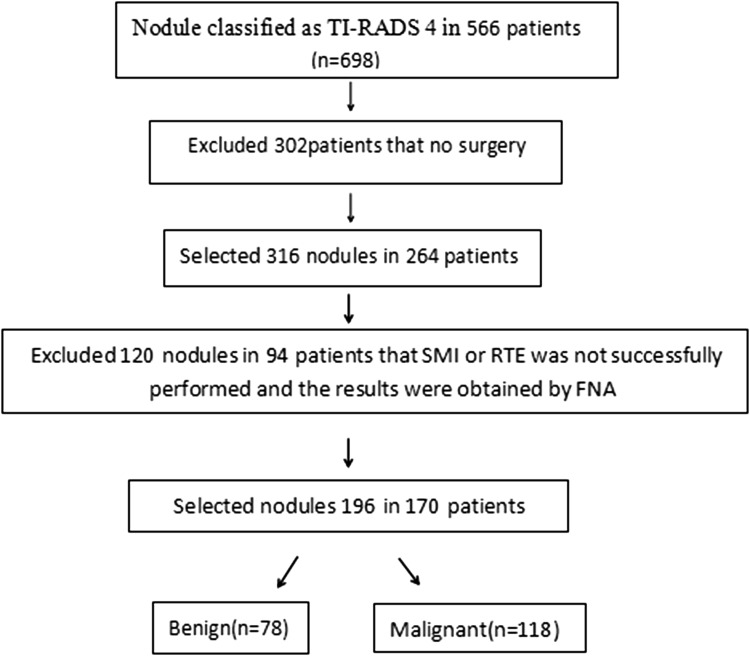
Inclusion criteria for the study. *FNA* fine-needle aspiration, *SMI* superb microvascular imaging, *RTE* real-time elastography

This study was performed at our hospital from January 2016 to April 2018 and examined 698 thyroid nodules in 566 consecutive patients that were classified as TI-RADS category 4. Of these, 302 patients were excluded from the study because they did not undergo surgery in our hospital. A total of 316 thyroid nodules in 264 patients was diagnosed based on histopathological results from surgical excision or fine-needle aspiration. Of these, 120 cases thyroid nodules in 94 patients were excluded because elastography imaging could not be successfully performed and the results were obtained by fine needle puncture. Thyroid nodules that met the following criteria were included (Fig. 3): (a) those meeting the criteria for TI-RADS category 4; (b) all patients with complete data, including US indicators and pathological findings; (c) all nodules, in which RTE and SMI were successfully implemented; and (d) all thyroid nodules that were not subjected to minimally invasive surgery prior to US examination (such as puncture and ablation), because these operations can affect the evaluation of nodules by RTE and SMI. Finally, 196 solid thyroid nodules in 170 patients were included in this study. This group comprised 112 women and 58 men. Pathological results of all nodules were obtained through surgery (*n* = 196).

All patients, respectively, underwent three modes of US imaging with a high-frequency transducer (L14-5, Aplio 500) before surgery. The patients were maintained in the supine position, with the head tilted backward to fully expose the neck. The patients were asked to hold their breath and avoid swallowing. First, conventional US examination was performed to observe the ultrasonographic features of the thyroid nodules. According to the TI-RADS classification system, five features of each nodule were assessed, including components, echogenicity, shapes, margin, and calcification. Each feature was assigned a corresponding score, and then the nodules were assigned different TI-RADS classifications according to their total scores. Nodules with scores ranging from 4 to 6 were classified as TI-RADS category 4, suggesting a moderate suspicion of malignancy.

### Superb microvascular imaging

After conventional US examination, SMI was routinely performed by the same ultrasonographer who executed the conventional US. The SMI has two modes—monochrome and color. Because the former has greater sensitivity [12–14], we selected the monochrome SMI in our study. A double-screen function, which allowed simultaneous visualization of gray scale imaging and SMI, was used when vessels were located. During the imaging process, the patients were asked to breathe quietly and avoid swallowing, so as to reduce interference. In addition, the examiner used a multi-directional and multi-angle lateral motion probe to carefully observe the distribution of blood flow in the nodule. Once the most abundant and clearest section of the blood flow signal was identified, the nodule blood supply characteristics and blood flow distribution patterns were recorded. A semi-quantitative method was proposed by Kim et al. [15], in which vascularity was classified as four patterns: type I, no credible vascularity (defined as no perivascular or dotted perinodular flow on < 25% of the nodule’s circumference and without any internal flow); type II, peripheral vascularity defined as surrounding > 25% of the nodule’s circumference without any internal flow; type III, dominant internal blood flow (in the solid nodule, defined as an increase in the blood vessels which were curved, angled, spiral, or irregular); and type IV, mixed vascularity (defined as the presence of any intranodular flow and peripheral flow on > 25% of the nodule’s circumference).

### Real-time elastography

After SMI examination, real-time elastography was routinely performed by the same ultrasonographer who performed the US and SMI. All elastography images were obtained in longitudinal planes. During elastography, the probe was positioned perpendicular to the skin while applying pressure. Images were obtained by applying light repetitive compressions on the skin above the targeted thyroid nodule. A square region of interest (ROI), with the target nodule positioned at the center of the box, was set for elastography acquisition. The ROI needed to be set to include sufficient surrounding thyroid tissue because elasticity in this system is displayed relative to the average strain inside the ROI. Thus, the top of the ROI was set to include subcutaneous fat and the bottom of the ROI was set to include the pectoral muscle; the borders were set to more than 5 mm from the lesion^’^s boundary [16, 17]. The probe was maintained at the lesion site, while slight vibrations were induced. The pressure on the display device was controlled at an index of 3–4, with a clearly visible hardness at all levels of tissue, and continued for 3–4 s [18]. Malignant and benign thyroid nodules were diagnosed according to a 5-point scoring system with RTE based on the following color patterns: 0 points, for lesions with a cystic component showing red and blue or blue-green and red; 1 point, for lesions and surrounding tissue in an even green color; 2 points, for lesions in green and the surrounding area in blue; 3 points, for lesions in blue and green; and 4 points, for lesions completely colored in blue. For US elastography images, a score of 3 points and higher indicated a malignant nodule, while a score of less than 3 points suggested a benign nodule [19–21].

To avoid interobserver variability, two ultrasonographers (with 5 and 10 years of experience, respectively) who were blinded to any clinical information, recorded the sonographic features; if a disagreement existed between them, a third expert with 20 years of experience made the final determination.

### Statistical analysis

The Student’s *t* test was used to compare patient age between the malignant and benign nodule groups. A *χ*^2^ test was used to analyze the differences in the distributions of conventional sonographic features, elastic score features, and vascular features between the groups. To investigate whether RTE, SMI, and the combination of the two methods could facilitate the diagnoses of TI-RADS category 4 thyroid nodules, the diagnostic value of any methods were compared using receiver operating characteristic curve (ROC) analysis. The *Z* test was used to compare the area under the ROC (*A*_z_). Statistical significance was determined as *P* < 0.05 (2-sided). Statistical analyses were performed using the SPSS version 20.0 statistical package (IBM Corporation, Armonk, NY, USA).

## Results

### Pathological diagnosis

The pathology results of 196 TI-RADS category 4 thyroid nodules identified 118 malignant thyroid nodules and 78 benign nodules; the malignant nodules comprised the following: papillary carcinoma (*n* = 107), medullary carcinoma (*n* = 4), follicular carcinoma (*n* = 4), and undifferentiated carcinoma (*n* = 3). The benign nodules comprised the following: thyroid adenoma (*n* = 12), nodular goiter (*n* = 60), and 6 others (subacute thyroiditis, *n* = 5; Hashimoto’s thyroiditis, *n* = 1).

### Features of conventional US, RTE, and SMI associated with benign and malignant TI-RADS category 4 thyroid nodules

Conventional US features including solid, hypoechogenicity or marked echogenicity, an aspect ratio ≥ 1, and microcalcifications were observed significantly more often in malignant nodules than in benign nodules (all *P* < 0.001). Additionally, type III blood flow patterns (determined by SMI) and elastic scores of 3 and 4 were more significantly associated with malignant nodules than with benign nodules (all *P* < 0.001). The details were presented in Table 1.

**Table 1:** US, SMI and RTE Features according to Malignant and Benign

Feature	No. of benign nodules (*n* = 78)	No. of malignant nodules (*n* = 118)	*P* value*
Components			0.002
Cystic (*n* = 0)			
Sponge-like (*n* = 0)			
Mixed (*n* = 21)	15 (71.4)	6 (28.6)	
Solid (*n* = 175)	63 (36.0)	112 (64.0)	
Echogenicity			< 0.001
Anechogenicity (*n* = 0)			
Iso- or hyperechogenicity (*n* = 18)	11 (61.1)	7 (36.8)	
Hypoechogenicity (*n* = 62)	36 (58.1)	26 (41.9)	
Marked hypoechogenicity (*n* = 116)	31 (26.7)	85 (73.3)	
Shape			0.011
Wider than tall (*n* = 114)	54 (47.4)	60 (52.6)	
Taller than wide (*n* = 82)	24 (29.3)	58 (70.7)	
Margin			0.873
Well defined (*n* = 30)	14 (46.7)	16 (53.3)	
Poorly defined (*n* = 148)	57 (38.5)	91 (61.5)	
Irregularity or lobuling (*n* = 5)	2 (40.0)	3 (60.0)	
Extracapsular spread (*n* = 17)	5 (38.5)	8 (61.5)	
Calcification			< 0.001
No calcification (*n* = 87)	44 (50.6)	43 (49.4)	
Macrocalcification (*n* = 30)	19 (63.3)	11 (36.7)	
Peripheral calcification (*n* = 9)	9 (100)	0 (0)	
Microcalcification (*n* = 57)	4 (7.0)	53 (93.0)	
Macro + Microcalcification (*n* = 13)	2 (15.4)	11 (84.6)	
SMI			< 0.001
I (*n* = 27)	22 (81.5)	5 (18.5)	
II (*n* = 49)	44 (89.8)	5 (10.2)	
III (*n* = 97)	5 (5.2)	92 (94.8)	
IV (*n* = 23)	7 (30.4)	16 (69.6)	
RTE			< 0.001
0 (*n* = 0)			
1 (*n* = 29)	29 (100)	0 (0)	
2 (*n* = 60)	37 (61.7)	23 (38.3)	
3 (*n* = 101)	12 (11.9)	89 (88.1)	
4 (*n* = 6)	0 (0)	6 (100)	

Unless otherwise indicated, data are numbers of nodules, and numbers in parentheses are percentages

*SMI* superb microvascular imaging, *RTE* real-time elastography

*P values were calculated using generalized estimating equation analysis

### Diagnostic performances of US, RTE, and SMI—which were applied alone and in combination—in evaluating TI-RADS category 4 nodules

**Fig. 4:**
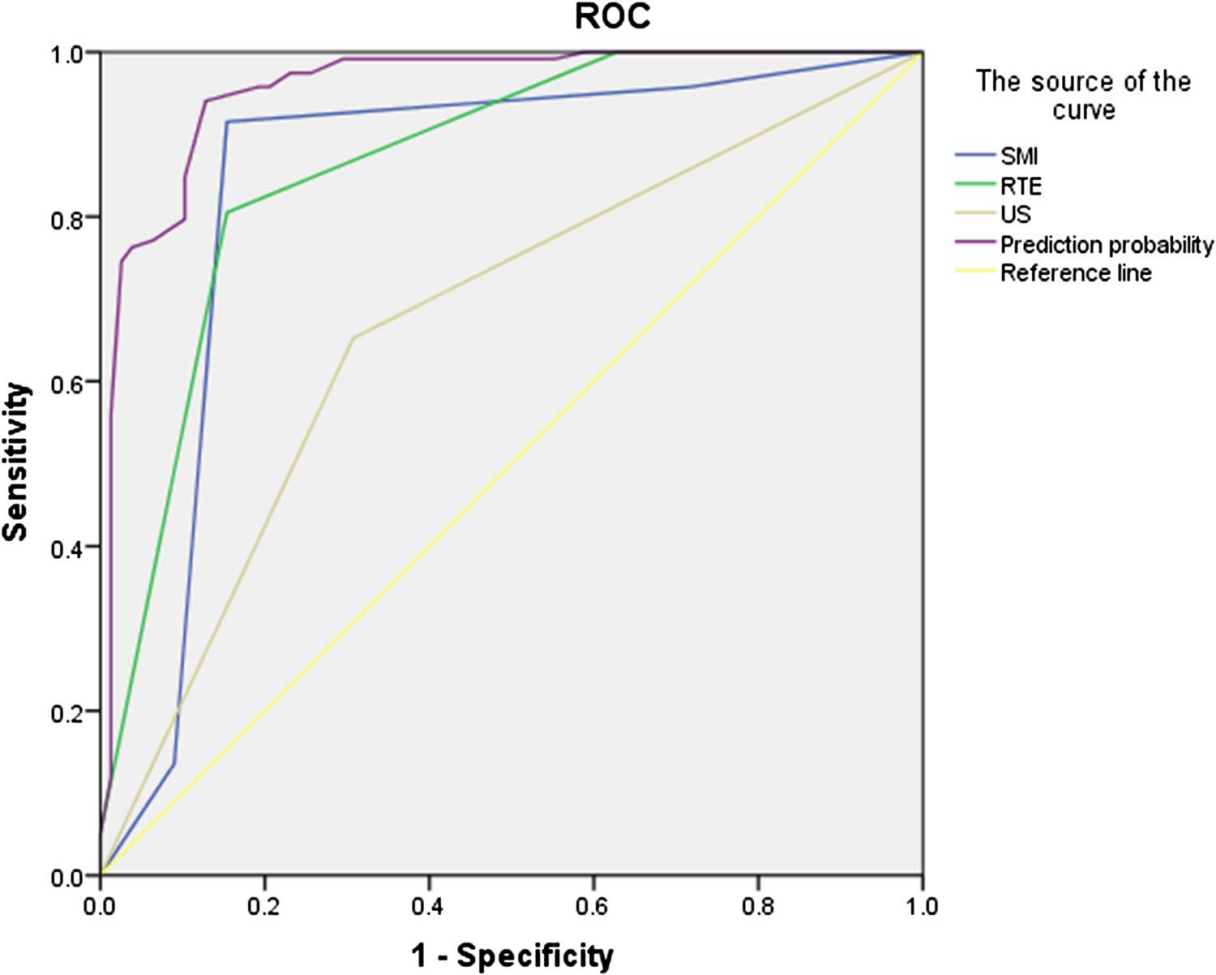
ROC curves of US, RTE and SMI in evaluating benign and malignant TI-RADS4 nodules. *SM I* superb microvascular imaging, *RTE* real-time elastography, *US* conventional ultrasound

In regard to the 196 nodules, the sensitivities, specificities, and accuracies of RTE and SMI were significantly higher than those of US. With regards to diagnosing TI-RADS category 4 nodules, RTE had a higher sensitivity than that of SMI, but the false positive rate of RTE was higher than that of SMI (Table 2). The accuracy of the combination of the three methods was significantly higher than that of each of the three methods separately (Table 2; Fig. 4).

**Table 2:** Diagnostic performance of US, RTE and SMI in 196 Nodules in 170 Patients

Methods	Sensitivity (%)	Specificity (%)	Accuracy (%)	FNR(%)	FPR(%)
US	65.25	69.23	66.84	34.75	30.77
RTE	80.51	84.62	82.14	19.49	15.38
SMI	77.97	93.59	84.18	22.03	6.41
Multimodal	94.08	87.18	91.33	6.93	12.82

*FNR* false negative rate, *FPR* false positive rate, *SMI* superb microvascular imaging, *RTE* real-time elastography, *US* conventional ultrasound

## Discussion

In our study, the sensitivity, specificity, and accuracy of US in the diagnosis of TI-RADS category 4 nodules were 65.25%, 69.23%, and 66.84%, respectively. Compared with previous studies, our results were lower than those of US in the diagnosis of benign and malignant all thyroid nodules [18]: this may be related to the nodules we studied. In the past, conventional US was used to evaluate all thyroid nodules, but the nodules of our study were classified as TI-RADS category 4. The benign and malignant characteristic of this type of nodules were often not obvious, and it was difficult to determine if they were benign or malignant by US alone. Therefore, it is necessary to establish a more effective method to accurately diagnose benign and malignant nodules of this type.

In this study, we evaluated TI-RADS 4 nodules using RTE. According to previous studies, a score of 3 was used as a diagnostic cut-off value. When the nodule elastic score was greater than or equal to 3, it was classified as malignant; those with a score less than 3, were classified as benign. Our results confirmed, that for the nodules of TI-RADS category 4 (which were difficult to identify using conventional US), the sensitivity, specificity, and accuracy of the RTE were 80.51%, 84.62%, and 82.14%, respectively. These results are comparable to those of Magri et al. [16, 17, 22, 23], who reported a sensitivity between 84.40 and 89.10% and a specificity between 70.00 and 85.30%. Our results showed that RTE has a higher sensitivity, specificity and accuracy than US in diagnosing TI-RADS 4 nodules, and the difference between the two methods was statistically significant (*P* < 0.05). However, it was clear that RTE had a relatively high false positive rate in diagnosing TI-RADS category 4 nodules. This may be attributed to multiple reasons [24–28]. For example, this study included some nodules with peripheral circular calcifications or internal semi-arc calcifications, in which the presence of calcifications may have increased the elasticity score. Additionally, to comprehensively evaluate the nodule, the other US features should be combined with that of RTE. On the other hand, some of the nodules were characterized by a mixed echo pattern. This included hypoechoic and slightly strong echo patterns and irregular forms, which are often caused by long-term fibrosis of the nodules, which also leads to increased elasticity scores.

When SMI was used to evaluate TI-RADS 4 nodules, it showed good diagnostic values. In terms of diagnosing benign and malignant TI-RADS 4 nodules, the sensitivity, specificity, and accuracy of SMI were 77.97%, 93.59%, and 84.18%, respectively. These results are consistent with those from a study by Kong et al. [29], who reported a sensitivity of 75.9%, and a specificity of 91.2%; other studies reported a sensitivity of 78.91% and a specificity of 85.1% [30]. Compared with RTE, SMI had a lower false positive rate. A possible reason for this is that the existence of ring and arc-calcifications may have led to a higher elasticity score and an inaccurate assessment. However, SMI can accurately assess the distribution pattern of blood flow within the nodule under these circumstances, thereby avoiding the effect of macro-calcifications.

Our results suggest that using both RTE and SMI, in conjunction with US, is a reliable method to differentiate between benign and malignant TI-RADS 4 nodules. When US was combined with the two other methods, the sensitivity, specificity, and accuracy in diagnosing TI-RADS 4 nodules were 94.08%, 87.18%, and 91.33%, respectively. These results were better than that of US, RTE, or SMI alone, and the difference was statistically significant (*P* < 0.05). Compared with US, RTE, and SMI, the accuracy of multimodal US imaging was increased, and the false negative rate decreased in terms of diagnosing benign and malignant TI-RADS 4 nodules. At the same time, the area under the ROC curve showed that multimodal US imaging was superior to the use of US, SMI, or RTE alone in the evaluation of benign and malignant TI-RADS 4 nodules (*P* < 0.001) (Fig. 4).

The above results may be related to the following reasons. First, in regard to the combination of the three methods, SMI helped compensate for the disadvantages of RTE in situations of calcification, depth, and trachea. This is because during elastography imaging, if the location was too deep, too closes to the trachea, or if the nodules presented with peripheral annular calcifications, those factors affected the elasticity score. On the other hand, for some smaller malignant lesions, the blood distribution pattern of SMI may show a type I or II pattern, leading to a false negative result. However, RTE was able to obtain information on the “hardness” of nodules through an elasticity score and achieve accurate diagnoses.

This study did have some limitations. First, the number of nodules we studied was small. Second, the pathological types of the nodules were relatively simple. The malignant nodules comprised primarily papillary carcinoma, with only four medullary carcinomas, four follicular carcinomas, and three undifferentiated carcinomas. Third, the assessments of RTE and SMI were not grouped according to nodular size. In future studies, we plan to collect more data, classify nodules according to size, and evaluate the diagnostic performances of SMI and RTE within the different groups of nodules.

The results of this study suggest that the application of RTE and SMI may help compensate in areas in which conventional US may be deficient in assessing the TI-RADS category 4 nodules; thus, multimodal US imaging—using the three methods—may provide more comprehensive information regarding the nodules, facilitating more accurate diagnoses. In conclusion, multimodal US imaging is beneficial in assessing TI-RADS category 4 nodules.
